# Sensorineural Hearing Loss after Magnetic Resonance Imaging

**DOI:** 10.1155/2013/510258

**Published:** 2013-06-17

**Authors:** Abolfazl Mollasadeghi, Amir Houshang Mehrparvar, Saeid Atighechi, Mohammad Hossein Davari, Pedram Shokouh, Mehrdad Mostaghaci, Maryam Bahaloo

**Affiliations:** ^1^Industrial Diseases Research Center, Department of Occupational Medicine, Shahid Sadoughi University of Medical Sciences, Yazd 89138-14389, Iran; ^2^Department of Occupational Medicine, Shahid Sadoughi University of Medical Sciences, Yazd 89138-14389, Iran; ^3^Department of Otolaryngology, Shahid Sadoughi University of Medical Sciences, Yazd 89138-14389, Iran; ^4^Cardiovascular Research Center, Isfahan Cardiovascular Research Institute, Isfahan University of Medical Sciences, Isfahan 81465-1148, Iran

## Abstract

Magnetic resonance imaging (MRI) devices produce noise, which may affect patient's or operators' hearing. Some cases of hearing impairment after MRI procedure have been reported with different patterns (temporary or permanent, unilateral or bilateral, with or without other symptoms like tinnitus). In this report, a case of bilateral sensorineural hearing loss in an otherwise healthy patient underwent brain MRI was described. The patient's hearing loss was accompanied with tinnitus and was not improved after 3 months of followup.

## 1. Introduction

Magnetic resonance imaging (MRI) usage as a diagnostic method is progressively increasing. Besides the advantages, MRI devices produce noise which may be hazardous for both operators and patients. It may cause such adverse effects as simple annoyance, anxiety, difficulty in verbal communication, changes in blood pressure and pulse rate, and temporary or permanent hearing threshold shift [1, 2].

The main source of noise during MRI procedure is vibration produced by gradient magnetic field which reaches the auditory system. Despite substantial progressions in designing and the introduction of gradient coil, the need for high-speed MRI devices has led to the production of loud noise-generating devices [3].

## 2. Case Presentation

Our patient was a 29-year-old man with refractory headaches who has not responded to standard treatments; therefore, a brain MRI was indicated for him. The MRI was performed using a 1.5 Tesla Magnetom, Avanto device (Siemens, Germany). During the 25-minute procedure, he did not use hearing protective equipment. One hour after the termination of the procedure, the patient felt tinnitus and hearing loss in his both ears. After two days, he was referred to our clinic without any considerable improvement in his symptoms. He did not complain of any other neurologic symptoms, such as blurred vision, sensory loss, or motor weakness.

No history of exposure to loud noise, ototoxic drugs or substances, head trauma, metabolic abnormalities, and familial deafness was observed. He did not report to have had tinnitus or hearing loss ever before. The patient had been working as a truck driver for five years and was not a smoker or alcohol consumer. 

**Figure 1:**
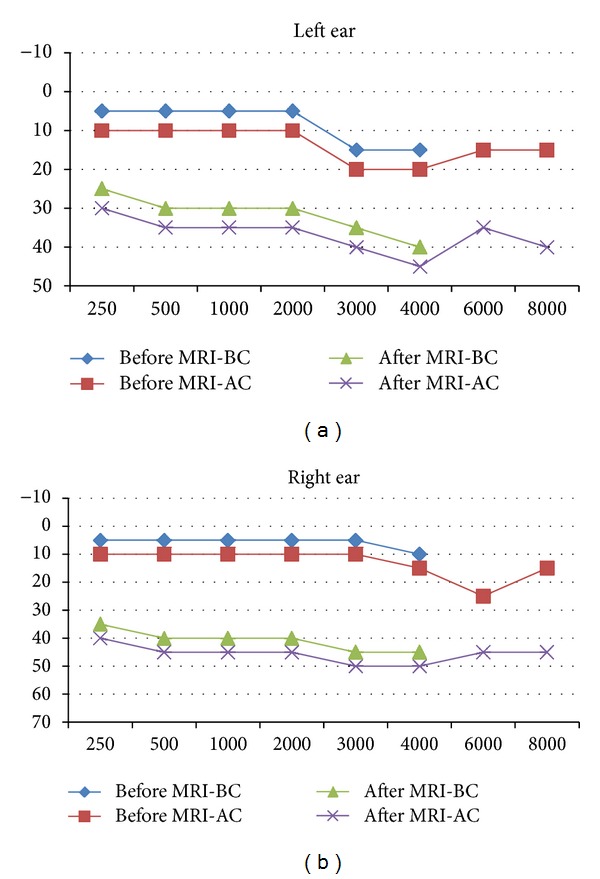
Patient's pure-tone audiogram recorded before and after magnetic resonance imaging (BC: bone conduction; AC: air conduction).

Of note, Rinne's test was positive which allowed us to rule out a conductive defect. In otoscopic evaluations, we failed to find any significant findings. Audiometry (device: AC40, Interacoustics), tympanometry (device: AZ26, Interacoustics), and otoacoustic emissions (OAEs) (device: Capella, Madsen) were also performed for him. As presented in Figure 1, pure-tone audiometry showed a flat sensorineural hearing loss. Tympanometry was normal and OAEs were not recorded. At 3 months of followup, he did not show any improvements in hearing thresholds.

## 3. Discussion

As stated before, the main noise-producing element in MRI systems is rapidly alternating currents within the gradient coil of the system [4]. Studies have shown that fast gradient echo pulse sequence may cause more noise during the procedure [5]. The most serious adverse effect of noise on humans is on hearing system, especially on the hair cells [6]. It has been shown that physical factors determining the extent of MRI noise-related hearing impairment include the duration of exposure and frequency and intensity of noise [1]. The frequency of noise created by MRI device, however, is usually below 4 KHz (mostly less than 2 KHz) [7, 8].

Price et al. have evaluated the noise produced by MRI scanners with different field strengths (from 0.2 to 3 Tesla). They reported the noise level to rise as the field strength increased (from 82.5 dB to 118.3 dB) [9]. However, the sound pressure produced by MRI devices seems not to be dependent merely on the field power as a range of 125.7 to 130.7 dB for 3-Tesla devices and a range of 101.8 to 111.7 dB for 1.5-Tesla devices have been reported [8]. 

Our patient suffered from a bilateral mild sensorineural hearing loss after 25 minutes of exposure to noise from a 1.5 Tesla MRI device, which did not improve after 3 months. Similarly, de Wilde et al. reported a case of hearing loss accompanied with severe headache and dizziness after a 0.5 Tesla MRI without hearing protection [10]. Govindaraju et al. also reported a case of unilateral hearing loss and tinnitus after a 3-Tesla MRI procedure. Inconsistent with our case, hearing loss was unilateral and improved after 3 days, although the tinnitus persisted [4].

According to what Price et al. [9] have recommended, it is not necessary for patients to wear hearing protection devices (HPDs) when being scanned by a ≤0.5-Tesla device; while in the case of operators, even these kinds of devices may be hazardous due to the long periods of exposure. In a case-control study, 43% of patients without HPDs suffered from transient mild hearing loss after 40 minutes of noise exposure in a 0.65-Tesla MRI device compared to only 10% of the control group with HPDs. Contrary to our observation, their hearing impairment was reversed after 15 minutes [11]. Using a MRI device similar to ours, Radomskij et al. compared hearing loss after MRI procedure between two groups of individuals with or without using of ear plugs. They found greater changes in OAEs among those without ear plugs, which remained in 68% of participants up to 10 minutes after the test [3].

The United Kingdom Medical Device Agency has set limits for MRI noise exposure at a threshold of 85 dB (averaged over 8 hours) for patients and 90 dB (averaged over 8 hours) for operators. The agency recommends using HPDs above these limits [12]. HPDs, if worn properly, can reduce noise level by 10–30 dB, which is usually adequate for protecting MRI operators and patients [11]. With respect to the United States National Institute for Occupational Safety and Health (NIOSH), the maximum permissible exposure time is halved by each 3 dB increase in sound pressure level [13]. Accordingly, when the level of noise reaches 100 dB the permissible exposure time reduces to 15 minutes [1, 9]. 

In conclusion, MRI devices produce noise that may impair the hearing system of operators and patients with such symptoms as tinnitus, headache, ear pain, and dizziness. Consequently, preventive measures should be applied in cases that are planned to undergo off-limit exposures.

## References

[1] McJury M, Shellock FG (2000). Auditory noise associated with MR procedures: a review. *Journal of Magnetic Resonance Imaging *.

[2] Bies DA, Hansen CH (2009). * Engineering noise control: theory and practice*.

[3] Radomskij P, Schmidt MA, Heron CW, Prasher D (2002). Effect of MRI noise on cochlear function. *The Lancet*.

[4] Govindaraju R, Omar R, Rajagopalan R, Norlisah R, Kwan-Hoong N (2011). Hearing loss after noise exposure. *Auris Nasus Larynx*.

[5] Ahmed S, Shellock FG (2001). Magnetic resonance imaging safety: implications for cardiovascular patients. *Journal of Cardiovascular Magnetic Resonance*.

[6] Baradarnfar MH, Karamifar K, Mehrparvar AH (2012). Amplitude changes in otoacoustic emissions after exposure to industrial noise. *Noise and Health*.

[7] Moelker A, Maas RAJJ, Lethimonnier F, Pattynama PM (2002). Interventional MR imaging at 1.5 T: quantification of sound exposure. *Radiology*.

[8] Hattori Y, Fukatsu H, Ishigaki T (2007). Measurement and evaluation of the acoustic noise of a 3 Tesla MR scanner. *Nagoya Journal of Medical Science*.

[9] Price DL, De Wilde JP, Papadaki AM, Curran JS, Kitney RI (2007). Investigation of acoustic noise on 15 MRI scanners from 0.2 T to 3 T. *Journal of Magnetic Resonance Imaging*.

[10] de Wilde JP, Grainger B, Price DL, Renaud C (2007). Magnetic resonance imaging safety issues including an analysis of recorded incidents within the UK. *Progress in Nuclear Magnetic Resonance Spectroscopy*.

[11] Brummett RE, Talbot JM, Charuhas P (1988). Potential hearing loss resulting from MR imaging. *Radiology*.

[12] Department of Health (1993). *Guidelines for Magnetic Resonance Diagnostic Equipment in Clinical Use*.

[13] NIOSH (1998). *Criteria for a Recommended Standard: Occupational Noise Exposure. Revised Criteria*.

